# Re-staging and follow-up of rectal cancer patients with MR imaging when “Watch-and-Wait” is an option: a practical guide

**DOI:** 10.1186/s13244-021-01055-w

**Published:** 2021-08-09

**Authors:** Inês Santiago, Bernardete Rodrigues, Maria Barata, Nuno Figueiredo, Laura Fernandez, Antonio Galzerano, Oriol Parés, Celso Matos

**Affiliations:** 1 Radiology Department, Champalimaud Foundation, Avenida Brasília, 1400-038 Lisbon, Portugal; 2 Nova Medical School, Campo Mártires da Pátria 130, 1169-056 Lisbon, Portugal; 3 Centro Hospitalar de Tondela-Viseu, EPE, Av. Rei Duarte, 3504-509 Viseu, Portugal; 4 Colorectal Surgery, Digestive Unit, Champalimaud Foundation, Avenida Brasília, 1400-038 Lisbon, Portugal; 5 Pathology Department, Champalimaud Foundation, Avenida Brasília, 1400-038 Lisbon, Portugal; 6 Radiation Oncology Department, Champalimaud Foundation, Avenida Brasília, 1400-038 Lisbon, Portugal

**Keywords:** Rectal cancer, Magnetic resonance imaging, Neoadjuvant therapy, Re-staging, “Watch-and-Wait”

## Abstract

In the past nearly 20 years, organ-sparing when no apparent viable tumour is present after neoadjuvant therapy has taken an increasingly relevant role in the therapeutic management of locally-advanced rectal cancer patients. The decision to include a patient or not in a “Watch-and-Wait” program relies mainly on endoscopic assessment by skilled surgeons, and MR imaging by experienced radiologists. Strict surveillance using the same modalities is required, given the chance of a local regrowth is of approximately 25–30%, almost always surgically salvageable if caught early. Local regrowths occur at the endoluminal aspect of the primary tumour bed in almost 90% of patients, but the rest are deep within it or outside the rectal wall, in which case detection relies solely on MR Imaging. In this educational review, we provide a practical guide for radiologists who are, or intend to be, involved in the re-staging and follow-up of rectal cancer patients in institutions with an established “Watch-and-Wait” program. First, we discuss patient preparation and MR imaging acquisition technique. Second, we focus on the re-staging MR imaging examination and review the imaging findings that allow us to assess response. Third, we focus on follow-up assessments of patients who defer surgery and confer about the early signs that may indicate a sustained/non-sustained complete response, a rectal/extra-rectal regrowth, and the particular prognosis of the “near-complete” responders. Finally, we discuss our proposed report template.

## Key points

- MR Imaging is one of the pillars for the selection and follow-up of patients when “Watch-and-Wait” is an option.
- Radiologists participating in “Watch-and-Wait” programs should be familiar with the imaging findings that indicate a poor/incomplete response, a complete response and a “near-complete” response.
- Given deep rectal and extra-rectal regrowth detection relies on MR imaging only, radiologists should combine a high index of suspicion with a high precision, to propel salvage surgery only when needed.

## Introduction

The concept of “locally-advanced rectal cancer” is intrinsically linked with a clinical indication for neoadjuvant therapy (NAT). It traditionally applies to all clinically staged T3/T4 and/or N+ tumours, although in the UK and other centres across Europe criteria may be more strict [1, 2]. NAT regimens were initially designed with the sole purpose of downsizing/downstaging tumours in order to increase the likelihood of an R0 resection and diminish the risk of local recurrence [3], but the 10–25% pathologic complete response (pCR) rates have led clinicians to question the utility of radical surgery itself in such cases [4]. In fact, the real pCR odds may be even higher, given most patients included in reported studies were operated at 6–8 weeks while pCR rates increase significantly when the interval to surgery is lengthened to > 12 weeks post-radiotherapy (RT) [5]. This means that if patients are re-staged and no signs of tumour persistence are present, deferral of surgery may be a reasonable option, and “Watch-and-Wait” (W&W) programs have grown increasingly available and popular during the past almost 20 years [4]. For willing and able patients, this decision is largely based on re-staging rectal endoscopy and MR imaging assessments [6]. Strict follow-up afterwards is founded on the same grounds [6]. The overall survival of clinical complete responders appears similar to that of patients who undergo surgery and have a pCR [7]. Furthermore, out of the approximately 30% clinical complete responders who later on present with tumour regrowth, 95% are salvageable [8]. However, patients with local regrowth may present with a higher rate of distant metastatic disease compared to sustained clinical complete responders [9]. Whether this phenomenon is a reflection of the tumour biology or a consequence of an uncontrolled primary is not yet known [9]. Magnetic resonance (MR) imaging, as part of response assessment, should aim to distinguish “true” complete responders from patients with persistent, even if subclinical, disease, who need surgery for cure as early as possible. This educational review has three purposes: the first is to provide radiologists with the main relevant information to re-stage rectal cancer patients after NAT based on standard MR imaging; the second is to guide them through W&W follow-up MR imaging assessments in order to detect pelvic regrowths /recurrences as early as possible; the third is to present and discuss our proposed report template.

## MR imaging preparation and acquisition technique

Before MR imaging acquisition, there are two preparatory steps which may significantly contribute to improve image quality. The first step is to ask patients to perform a small enema for rectal emptying before entering the MR equipment [10]. The second is to administer a spasmolytic agent such as butylscopolamine or glucagon before acquisition [10]. These two preparation steps are meant to minimise susceptibility artefacts due to air content and movement artefacts from peristalsis, respectively, given both adversely impact image interpretation, of diffusion-weighted imaging (DWI) in particular [10]. They are considered optional according to both ESGAR and SAR guidelines [11, 12]. When despite these measures patients still present with rectal air at tumour bed level and if they agree, a lubricated cannula can be inserted in the rectum for further emptying, and then removed before acquisition.

The use of endoluminal gel is not advocated routinely because distension of the rectum may result in misinterpretation of the distance between the tumour bed and circumferential resection margin (CRM), an essential re-staging information when surgery is a possibility [13]. If considered useful, gel should not exceed 60 ml to prevent excessive compression of the mesorectal fat [14].

Regarding technique, examinations should be performed on a 1.5T or 3T equipment using an external coil [11, 12]. 2D high-resolution T2-WI with a slice thickness $\le$3 mm should be acquired in sagittal, axial and coronal planes, the two latter angulated perpendicular and parallel to the long axis of the tumour, respectively [11, 12]. In low tumours, additional high-resolution T2-WI angulated perpendicular and parallel to the anal canal may be acquired to better assess its involvement [11, 12]. A DWI acquisition including a high b value (≥ 800) should also be acquired, preferably with the same orientation as the axial high resolution T2-WI to ease finding co-localisation [11, 12]. Intravenous paramagnetic contrast administration is not routinely recommended [11, 12] but it may be useful in particular situations such as pelvic sepsis or fistulisation. Detailed acquisition protocols for our 1.5T and 3T Ingenia Philips Healthcare®, Best, The Netherlands clinical scanners are provided in Table 1.

**Table 1:** Re-staging pelvic MR imaging acquisition parameters at 1.5T and 3T Ingenia Philips Healthcare®, Best, The Netherlands

	Oblique axial T2-W turbo spin-echo		Oblique coronal T2-W turbo spin-echo		Sagittal T2-W turbo spin-echo		Oblique axial single-shot spin-echo echo-planar diffusion		Axial T1-weighted gradient echo imaging	
	1.5T	3T	1.5T	3T	1.5T	3T	1.5T	3T	1.5T	3T
Echo time (msec)	115	105	115	105	105	100	92	95	10	15
Repetition time (msec)	4206	3943	4206	3943	2433	4672	6779	4140	683	734
Echo train length	17	19	17	19	17	17	–	–	5	7
Slice thickness (mm)	3	3	3	3	3	3	4	4	45	4
Gap (mm)	0	0	0	0	0	0	0	0	1	0.8
Matrix	400 x 333	400 x 259	400 x 333	400 x 259	252 × 223	252 x 237	76 × 65	80 × 65	376 x 390	404 × 415
Field-of-view (mm)	200 x 200	200 × 200	200 x 200	200 × 200	200 × 200	200 × 200	200 × 200	200 × 200	300 x 350	300 × 350
In-plane resolution (mm)	0.5 x 0.6	0.5 x 0.6	0.5 x 0.6	0.5 x 0.6	0.8 × 0.8	0.8 x 0.8	2.6 × 3.01	2.5 × 3.1	0.8 x 0.9	0.74 × 0.82
Signal averages	2	2	2	2	2	1	14	13	1	1
B value	–	–	–	–	–	–	1500	2000	–	–

Oblique axial scans are oriented perpendicularly to the long axis of the rectal wall at tumour location; ^+^Spectral pre-saturation with inversion recovery is utilised for fat saturation

## Re-staging after neoadjuvant therapy

The issue of when to first assess tumour response is controversial. While the rate of pCR may increase after 12 weeks post-RT [5] some surgical teams are reluctant to operate beyond 8 weeks due to concerns about radiation-induced pelvic fibrosis and related surgical complications [15], which implies identifying poor/incomplete responders early. To move the decision timepoint from 6–8 weeks to 10–12 weeks post-RT may have no impact on surgery-related morbidity or mortality [16], and the extended period of surveillance may allow the start of consolidation chemotherapy on metastatic high-risk patients that may benefit from total NAT.

Although clinical and laboratory evaluation is relevant, re-staging relies heavily on rectal endoscopy and MR imaging. If the two are to be performed on the same day and given endoscopy requires the insufflation of air into the rectum, to perform MR imaging first may be preferable in order to minimise air-induced susceptibility artefacts.

Endoscopic assessment [16] may be standardised according to a 5-point ordinal scale defined by the international W&W database consortium: responses may be graded, from best to worse response, as 0 (flat white scar with telangiectasia), 1 (shallow ulcer/red scar), 2 (ulcerated residual lesion or adenomatous residual mucosal abnormality), 3 (excavated ulcer with elevated edges) or 4 (persistent infiltrative lesion).

### To identify the incomplete/poor responders

**Fig. 1:**
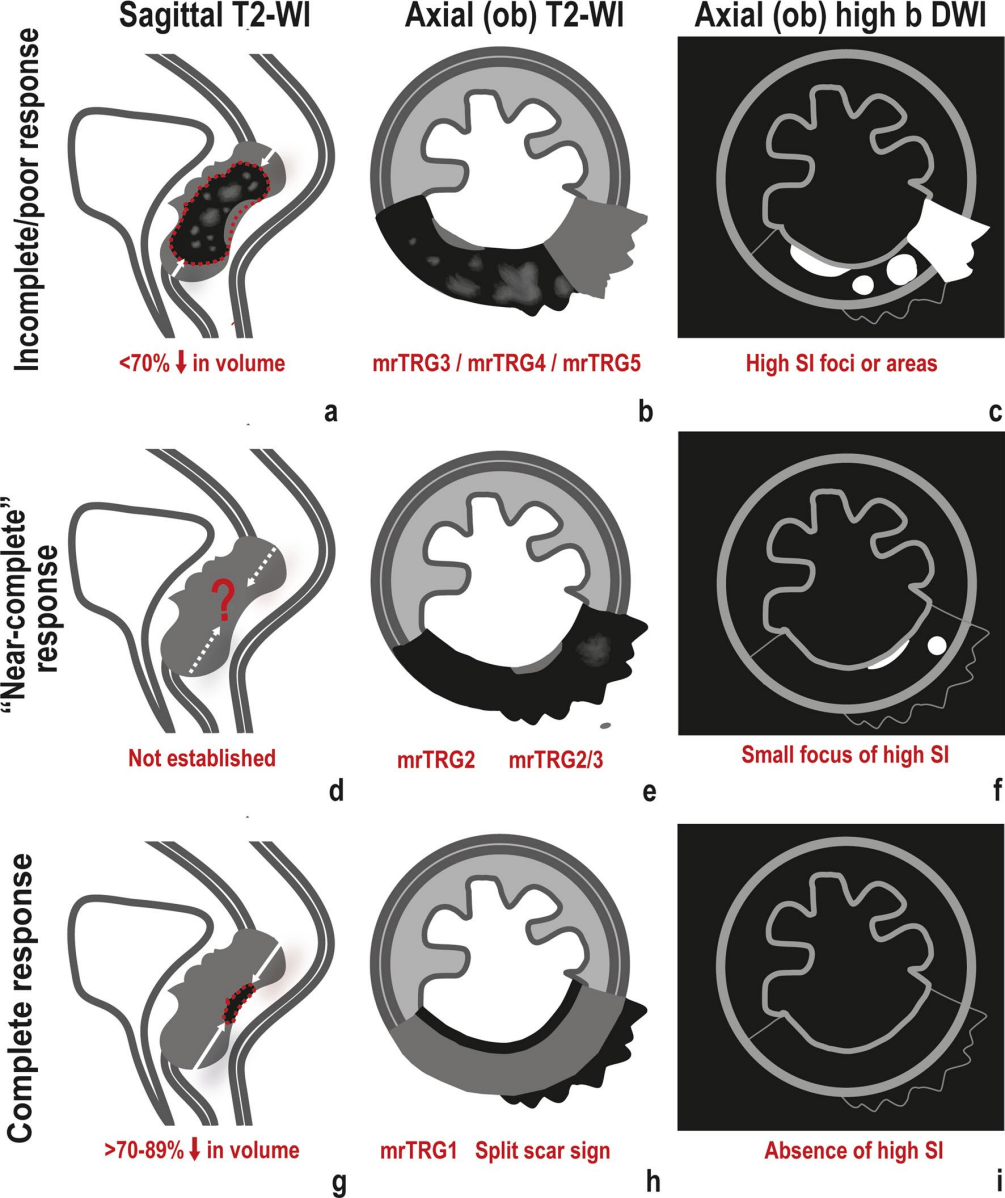
Primary tumour response. An incomplete/poor * response* to NAT may be characterised by (**a**) little reduction in tumour volume, < 70%. **b** mrTRG3 or higher, translating into the presence of residual intermediate “tumour” signal intensity on T2-WI; **c** Residual high signal intensity at tumour bed on DWI. “*Near-complete” responses* fall on the good response group and as such, a large reduction in tumour volume may be expected but a cutoff is not established (**d**). **e** On T2-WI, mrTRG2—scar with “dense fibrosis”—and mrTRG2/3, in which a tiny focus of residual intermediate signal may still be visible, are included; **f** Also, a small residual focus of high signal intensity on high b-value DWI may be admitted. *Complete responses* are characterised by a very large reduction in tumour size, > 70–89% (**g**). **h** on T2-WI, they may present as mrTRG1, characterised by the presence of a linear/crescenteric 1–2 mm hypointense scar at the endoluminal aspect of the tumour bed or normalisation of the rectal wall; they may also present with a positive split scar sign, which includes mrTRG1s and scars with an additional peri-rectal, usually irregular, layer of hypointense tissue, separated from the inner linear hypointense linear/crescentic scar by an intermediate signal intensity thickened and partially fibrotic *muscularis propria*; **i** No high signal intensity at tumour bed on DWI is expected

Approximately 20% of locally advanced rectal cancer patients show a poor response to neoadjuvant therapy, which may imply a shift in the neoadjuvant treatment scheme or early surgery [17, 18]. Poor responses generally present with endoscopic gradings of 3–4 and on MR imaging are characterised by (Fig. 1a–c):

1. Little reduction in tumour size

A tumour volume reduction below 70%, as based on slice-by-slice segmentation on T2-WI, is associated with a poor response [19–22]. Given tumour volume measurement is difficult and time-consuming, cranio-caudal length was suggested as an alternative [12]. Based on mrRECIST, less than 30% reduction in cranio-caudal tumour length is associated with a poor response [23]. However, this criteria was not validated prospectively [24].

2. MR Tumour regression grades (mrTRG) 3–5

MrTRG is an ordinal scale developed by Patel et al.to assess response on MR T2-WI in a parallel manner to the Mandard pathological TRG score [23, 25] and is presented in Table 2 [18].

**Table 2:** mrTRG and corresponding MR imaging findings [23]

mrTRG—Findings on MR imaging
1. Complete response (linear/crescentic 1–2 mm scar in mucosa or submucosa only, or normalisation of the rectal wall)
2. Good response (dense fibrosis; no obvious residual tumour, signifying minimal residual disease or no tumour)
3. Moderate response (> 50% fibrosis or mucin, and visible intermediate signal)
4. Slight response (little areas of fibrosis or mucin but mostly tumour)
5. No response (intermediate signal intensity, same appearances as original tumour/tumour regrowth)

There are multiple ordinal scales in the literature that represent variations or adaptations to it but in general, when T2-WI intermediate “tumour” signal predominates over hypointense fibrosis or clear hyperintense “acellular mucin” (mrTRG4-5, mrTRG5 hardly ever being observed in our experience), a poor response is assumed, and the 3-year disease free survival is 21% inferior compared to favourable mrTRG1-3s [23]. mrTRG3 may fall on the “good side” regarding long-term outcome [26] but the likelihood of a pCR or cCR when T2-intermediate clearly “tumoural” signal remains after neoadjuvant treatment is low, and it is therefore considered a sign of an incomplete response [27].

3. Significant restriction to diffusion on Diffusion-weighted Imaging (DWI)

DWI is highly sensitive to susceptibility artefacts and dependent on good acquisition technique. Good patient preparation is essential as well as adequate training in image reading. T2-shine through effects from luminal content, which will present with high signal intensity on high b value images and high signal intensity in the ADC map, should not to be mistaken for true restriction, which will present with high signal intensity on high b value images and low signal intensity in the ADC map; or with dense fibrosis, which will present with low signal intensity in both high b value images and ADC map. While on T2-WI mrTRG the proportion of intermediate “tumour” signal at tumour bed is taken into consideration, in most studies focusing on response assessment using DWI, assessment is binary—restriction indicates viable tumour whereas its absence suggests a complete response [28–31]. Also, DWI performance is generally evaluated in addition to T2-WI, and combined reading appears to perform significantly better for most readers [28–31]. Diffusion restriction may also be present in radiation-induced proctitis or hemorrhage/inflammation post-biopsy and when these procedures are performed before MR imaging, the radiologist should be notified. Also, restriction may be absent in incomplete responses, and according to Lambregts et al., in clear T2-WI intermediate-signal residual masses and in T2-hypointense “fibrotic” circumferential tumour scars, irrespective of DWI findings, the likelihood of viable tumour is very high (≥ 80%) [32].

Mucinous tumours have an intrinsically higher ADC compared to non-mucinous tumours, making response assessment based on DWI more difficult and less reliable [33].

**Fig. 2:**
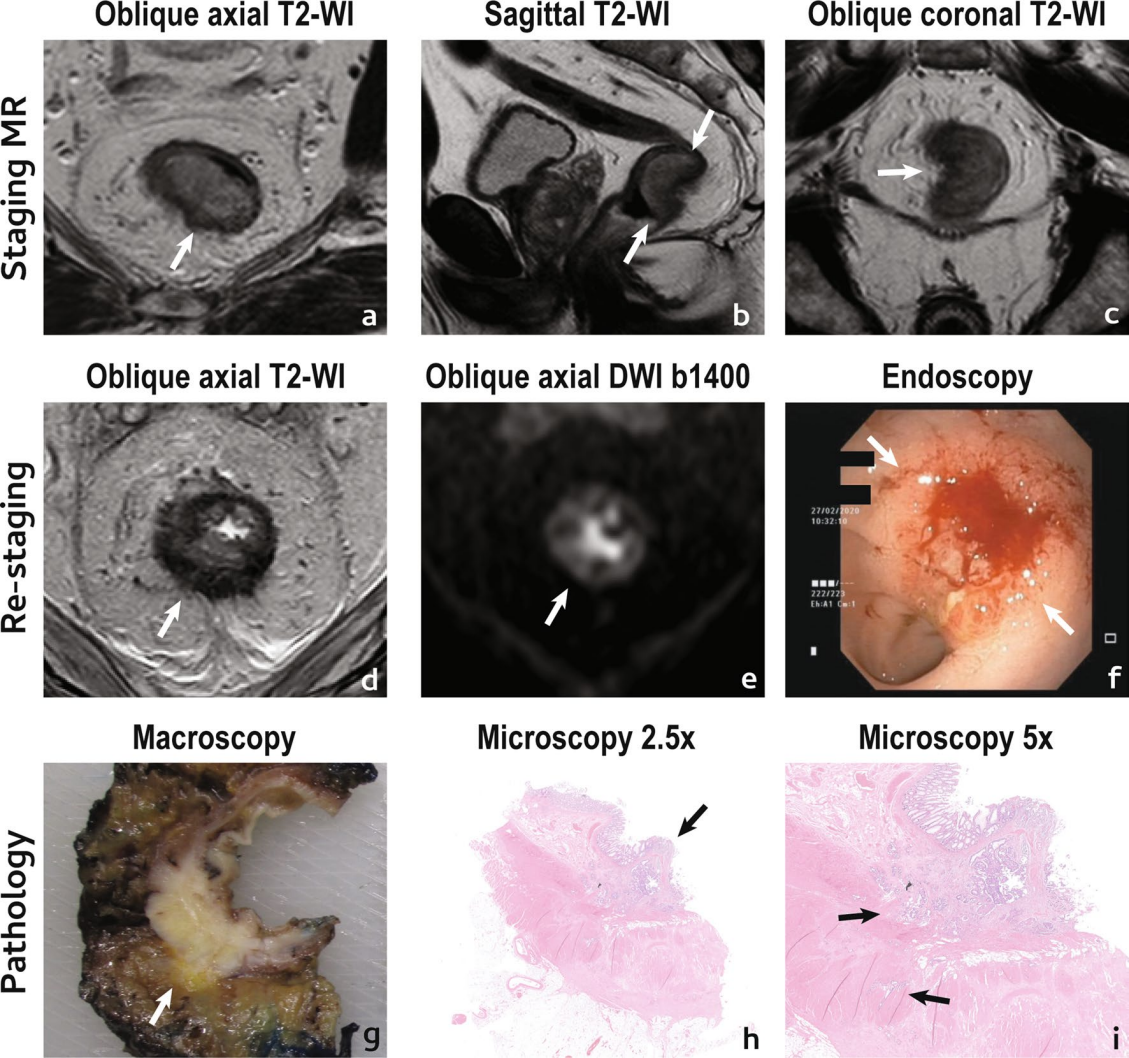
Incomplete/poor tumour response. A 75 year-old male presented with a low mrT3a mrN0 EMVI + (not shown) CRM-rectal cancer (arrows in **a**–**c**). He underwent NAT and was re-staged at 12 weeks with MR imaging (**d**, **e**) and endoscopy (**f**). Reduction in size was estimated as < 50%, as may be inferred in **d** vs **a**. On T2-WI, the tumour scar was composed largely of intermediate signal intensity tissue (**d**), classified as mrTRG4 with absent split scar sign. On high b value DWI (**e**), a thick layer of high signal intensity was apparent at the endoluminal aspect of the tumour bed and a persistent infiltrative lesion was visible on endoscopy (between arrows in **f**). Patient underwent surgery and specimen was staged as a ypT3 (extension into mesorectal fat visible at macroscopy in **g**) N1c (not shown) TRG3 R0. At microscopy, viable tumour was predominantly mucosal/submucosal (arrow in **h**) but there were niches of viable tumour cells within the muscularis propria (arrows in **i**) and also at perirectal fat (not shown)

An example of a poor tumour response is given in Fig. 2.

**Fig. 3:**
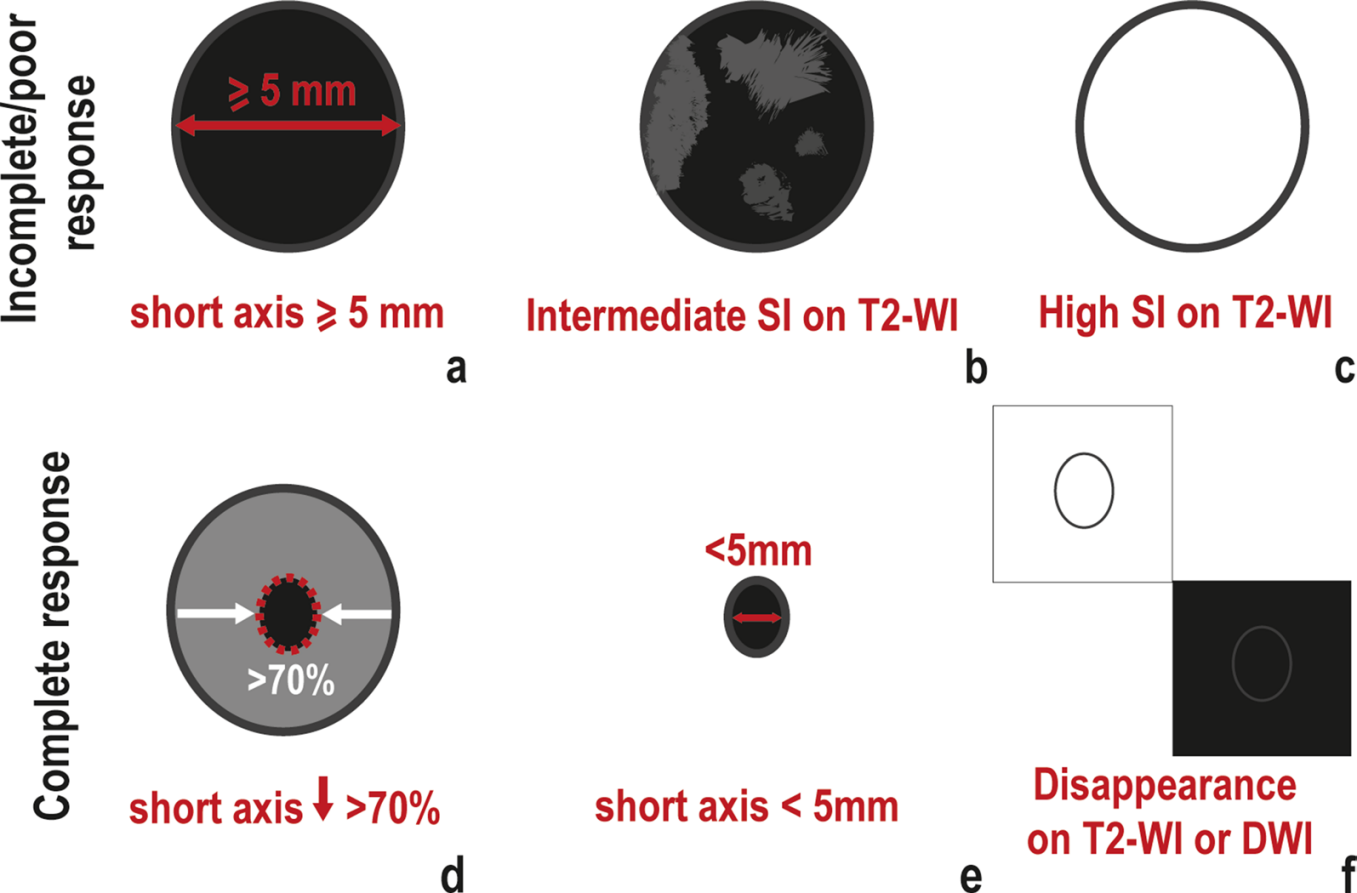
Mesorectal lymph nodes. An incomplete/poor lymph node response to NAT may be characterised by (**a**) a short axis ≧5 mm after NAT; **b** residual intermediate “tumour” signal intensity or heterogeneity on T2-WI; **c** Persistent high signal intensity “mucin” on T2-WI. When a lymph node presents with a complete response after NAT, a reduction in short axis superior to 70% at re-staging T2-WI may be observed (**d**). Also, according to ESGAR guidelines, lymph nodes with short axis < 5 mm on re-staging T2-WI (**e**) should be considered negative. The positive predictive value for a complete response in lymph nodes that disappear on T2-WI or DWI (**f**) is close to 100%. “Near-complete” responders should have no signs of persistent disease in lymph nodes and as such criteria are the same as for complete responders

With respect to mesorectal lymph nodes, lymph nodes with incomplete/poor response usually present with (Fig. 3a–c):

1. *Short axis* ≥ *5 mm*, which may be associated with a likelihood of residual disease up to 63% [12, 34].
2. *Residual intermediate “tumour” signal intensity or heterogeneous signal intensity on T2-WI*, which usually represents residual macroscopic tumour [34].

Residual high signal intensity on high b value DWI with low ADC does not aid in the identification of viable tumour in lymph nodes given it may be observed in both lymphoid and tumour tissue.

3. *Residual high “mucin” signal on high b-value T2-WI — *Mucinous tumours with lymph node involvement present with a higher frequency of residual viable tumour after neoadjuvant therapy and as such, lymph nodes with visible “mucin” high signal intensity on T2-WI at re-staging MR imaging are frequently positive [35].

**Fig. 4:**
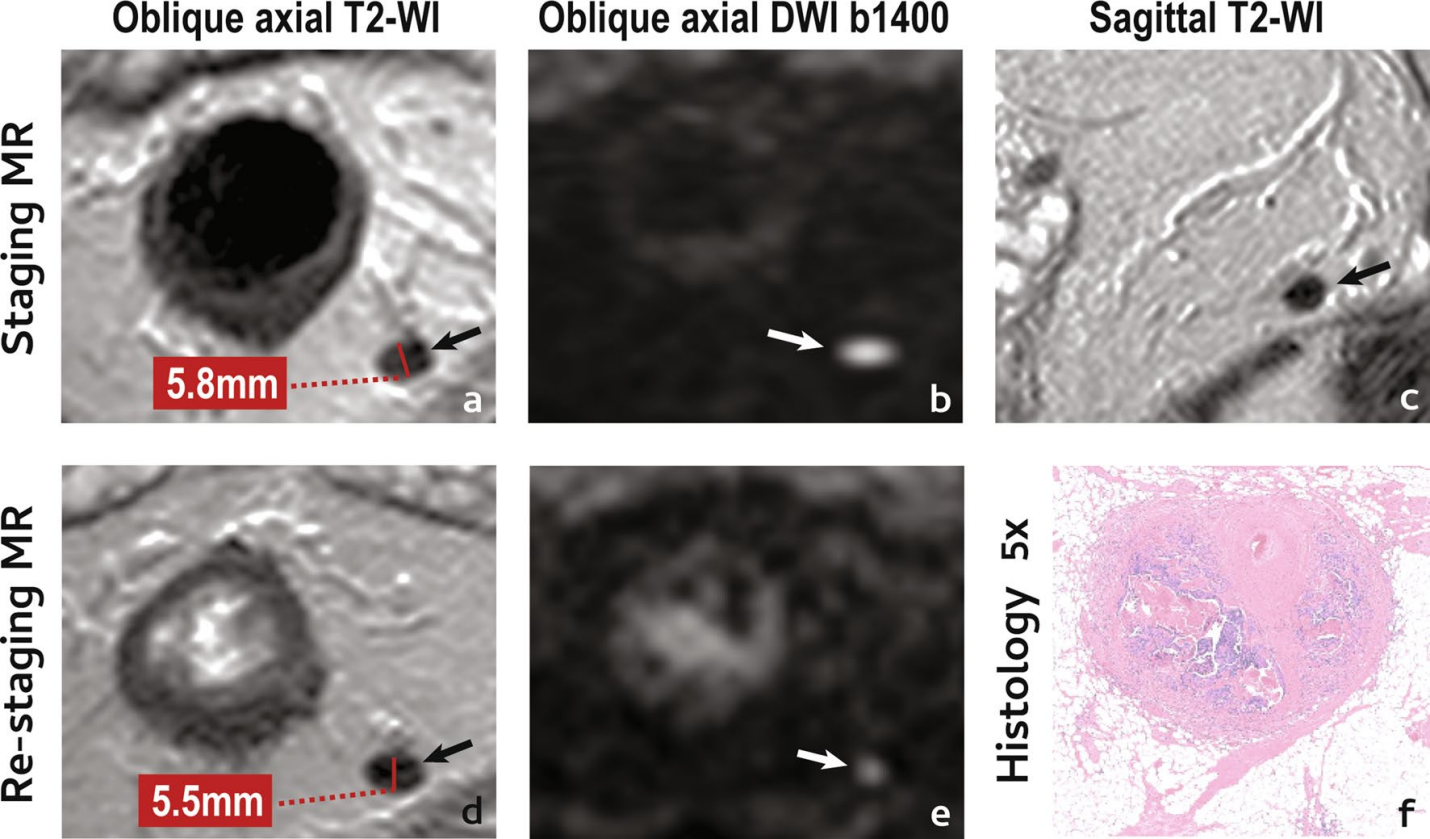
Incomplete/poor lymph node response. A 65 year-old male presented with a low mrT3b (not shown) mrN1 (arrow in **a**–**c**) EMVI− CRM+ rectal cancer. He underwent NAT and was re-staged. Lymph node reduction in size was practically inexistent and post-NAT, short axis was > 5 mm (**d**). On T2-WI, the lymph node still looked heterogeneous, with areas of intermediate signal intensity (**d**). High signal intensity on high b value images was less but still present (**e**) (please note that visibility on high b value post-NAT imaging does not exclude good or even complete response). Patient underwent surgery and no residual lymphoid tissue was found within the lymph node in the pathology specimen, only tumour surrounded by fibrosis, adjacent to a vessel (**f**)—patient was therefore staged as a ypT3 N1c TRG3 R0

An example of a poor lymph node response is given in Fig. 4.

Involvement of lateral pelvic sidewall lymph nodes is more likely to occur in tumours located at the level or below the peritoneal reflection, particularly if ≥ T3 [36]. The lower the location of the primary lesion, the higher the risk and in tumours < 4 cm from the anal verge, it may exceed 30% [36]. Lymph nodes with mixed signal intensity, irregular borders or short axis ≧ 5, 7 or 8 mm (different cutoffs are considered in different studies) on staging examinations were associated with a higher likelihood of harbouring metastasis [37, 38]. On re-staging MR imaging, criteria associated with residual tumour may be:

1. *Size reduction* < *33%* between pre and post-neoadjuvant treatment [31].
2. *Short axis* > *5 mm* on post-neoadjuvant therapy MR [38].
3. *Residual “tumour” signal intensity or heterogeneous signal intensity on T2-W,I* just the same as applied to mesorectal lymph nodes.

Regarding response of extramural venous invasion (EMVI) to neoadjuvant therapy, Chand et al.have established a specific TRG score for EMVI (mr-vTRG) on T2-WI and concluded grades 4 (< 25% fibrosis) and 5 (minimal fibrosis) were associated with higher local recurrence rates (44%) and lower disease-free survival (46%) compared to grades 1–3 (50% fibrosis or more)—9% local recurrence and 88% 3-year disease-free survival [39]. In our experience assessment of percentage of conversion to fibrosis may be difficult but indeed residual intermediate “tumour” signal intensity within EMVI after neoadjuvant therapy should signify viable tumour and a consequent poor or incomplete response,  remaining restriction to diffusion supporting it. Although we found no data on the response of extranodal tumour deposits, similar criteria may apply.

Given incomplete/poor responders may undergo early surgery, it is particularly important that their identification is followed by detailed information on residual tumour location and relations. The most important of all items is the re-evaluation of the circumferential resection margin (CRM) given a clearance of the margin on re-staging high-resolution T2-WI has a positive predictive value of up to 90% for a clear margin at pathology, which may justify a shift towards less mutilating surgery [40–42]. On the other hand, if the margin is reached by dense hypointense fibrosis, the likelihood of tumour at pathology is lower than when it is reached by intermediate “tumour” signal intensity but is still significant, and as such it should be considered involved [40–42].

### To identify the complete responders

Approximately 10–25% of patients with locally-advanced rectal cancer undergoing NAT prior to surgery achieve a pCR [4], escalating to even higher rates when more intense RT and/or chemotherapy regimens are employed [4].

There are some factors at staging MR that may be associated with a higher likelihood of a pCR, namely tumour length < 4 cm, tumour circumference < 180º, distance to anal verge < 45 mm and mrT stage ≤ 3 [43, 44]. Also, pCR rate increases with increasing interval to surgery to > 12 weeks [5], which means signs of a clinical complete response (cCR) on re-staging MR imaging are expected more often with longer intervals as well. Clinical complete response is characterised by a flat white scar with telangiectasia on endoscopy (endoscopy 0). At re-staging MR imaging the following are expected regarding the primary tumour (Fig. 1g–i):

1. Very large reduction in tumour size

Volume reduction > 70–89% at T2-WI (T2-hypointense “fibrosis” included in the measurements) and > 95–98% at DWI (only high signal intensity on high b value images measured) associate with a complete response to treatment [45–47].

2. mrTRG 1

Conversion to a linear/crescentic, 1–2 mm scar in the mucosa or submucosa or normalisation of the rectal all have very high specificity for complete response, in the range of 92–98% [48].

3. Positive split scar sign

**Fig. 5:**
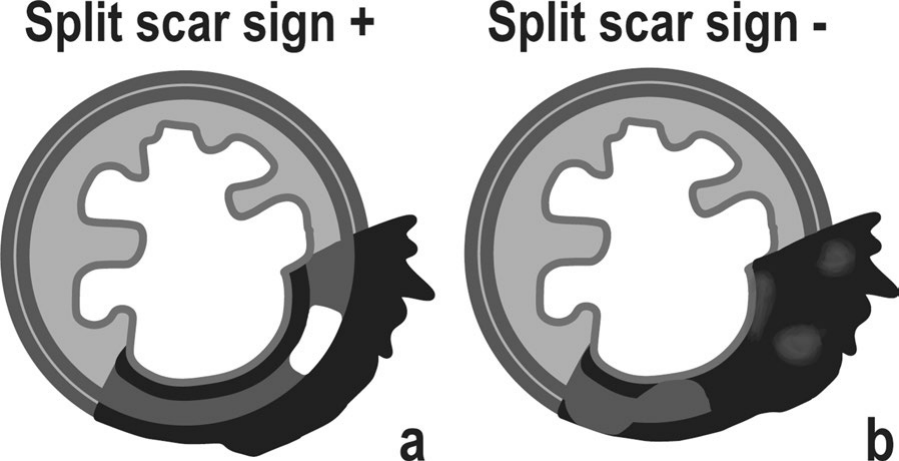
The split scar sign is considered present or positive (**a**) when a thin 1–2 mm regular layer of hypointense “fibrosis” is present at the endoluminal aspect of the tumour bed (corresponding to the fibrosed submucosa), underlined by homogeneous intermediate signal (corresponding to a thickened and partially fibrotic muscularis propria), covered or not by a usually irregular hypointense layer of perirectal fibrosis. In mucinous or mucin-degenerated tumours, the middle intermediate signal intensity layer may be replaced with homogeneously high signal intensity, corresponding to mucin pooling. Whenever the tumour bed is not “organised” in such a layered manner (such as with full thickness “black” scars) or whenever it is but the inner and/or outer “fibrotic walls” are focally breached, the sign should be considered absent/negative (**b**) [26]

The split scar sign has a very high specificity (97%) and positive predictive value (93/94%) for a sustained complete response [46], but it was not yet validated prospectively. It may be found on high resolution T2-WI and is characterised by an organised layered morphologic pattern of the tumour bed after neoadjuvant therapy, composed of an inner thin and regular hypointense band corresponding to the fibrosed submucosa; an intermediate signal intensity layer immediately underneath it, corresponding to a thickened and partially fibrosed *muscularis propria*; and an outer, irregular, hypointense layer of mesorectal fibrosis, which may be absent and usually is in staged ≦ T2 tumours [49]. The split scar sign is explained in greater detail in Fig. 5.

4. Absence of high signal intensity on high b value DWI

Complete response on DWI is supported by the absence of high signal intensity at high b-value DWI images (using normal rectum as reference) [28–31] and it may be particularly valuable in small, subcircunferencial scars [32], whereas as previously discussed, in thick, circumferential T2-hypointese “fibrotic” responses, the likelihood of incomplete response is high even if DWI is negative [32].

**Fig. 6:**
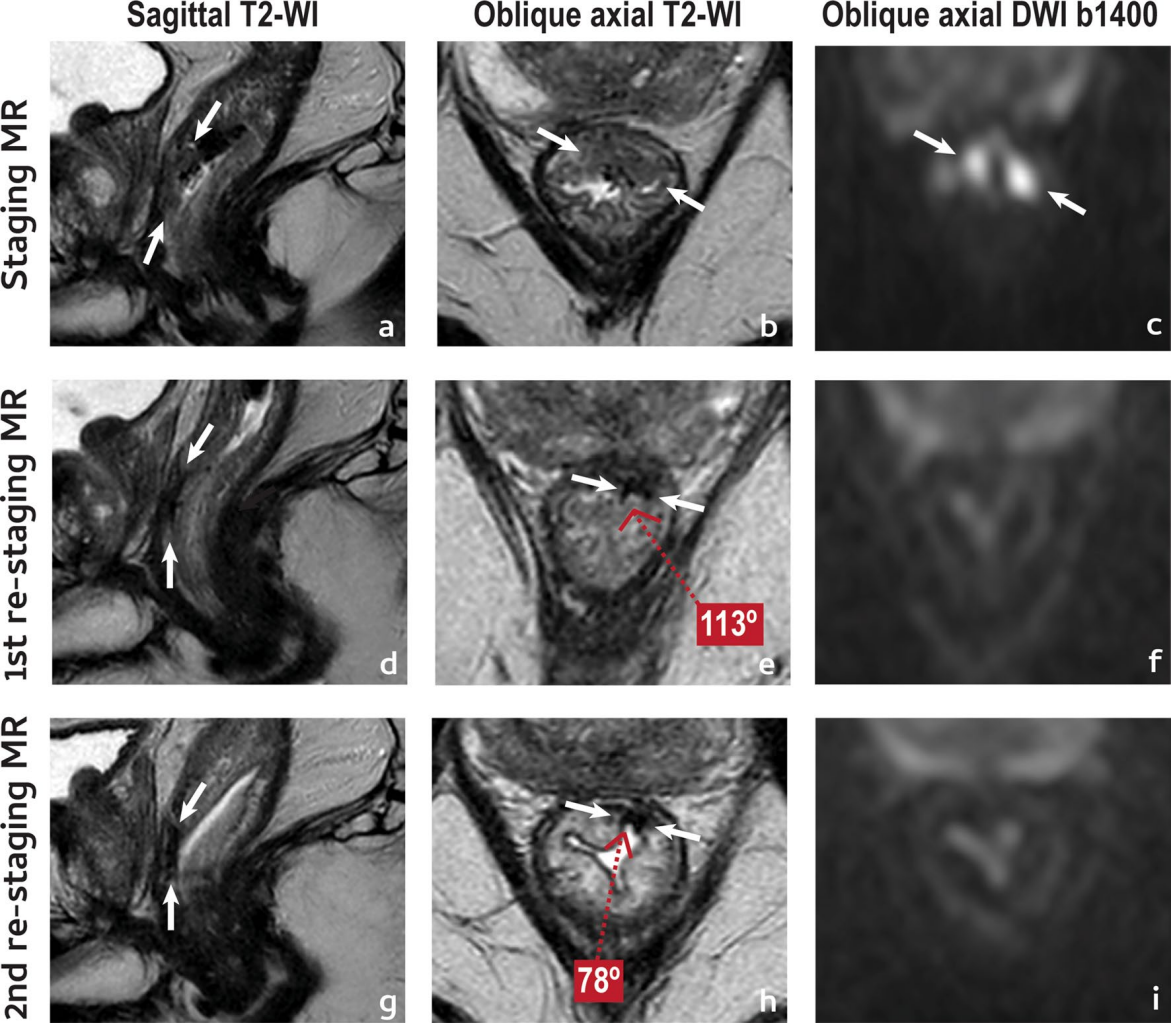
Complete tumour response. A 61 year-old male presented with a low anterior mrT2 (between arrows in **a**–**c**) mrN1a (not shown) EMVI− rectal cancer. He underwent NAT and was re-staged at 11 weeks (**d**–**f**). Reduction in tumour size was considerable, > 80% (**d, e**). On T2-WI, the tumour was reduced to a thin, crescentic, endoluminal hypointense “fibrotic” scar (mrTRG1, split scar sign+) (**e**). On high b value DWI, no high signal intensity was visible (**f**). Notice how the scar “curled-in” between first and second assessment (**e**, **h**, respectively), reducing its depth angle significantly (− 35°). Patient is currently on W&W with no signs of disease recurrence at 1 year

An example of a complete tumour response is given in Fig. 6.

With respect to mesorectal lymph nodes, a complete response may present with (Fig. 3d–f):

1. *Significant size reduction or disappearance on T2-WI.* A ≥ 70% short axis reduction or disappearance of the LN on T2-WI may indicate ypN0 status in 100% of cases [34] and according to ESGAR guidelines, LNs < 5 mm after neoadjuvant therapy should be assumed as negative [12].
2. *Disappearance on DWI*—Absence of visible lymph nodes in high b value DWI may be a reliable predictor of ypN0 status [50] but if high signal persists, a complete response may not be excluded.

**Fig. 7:**
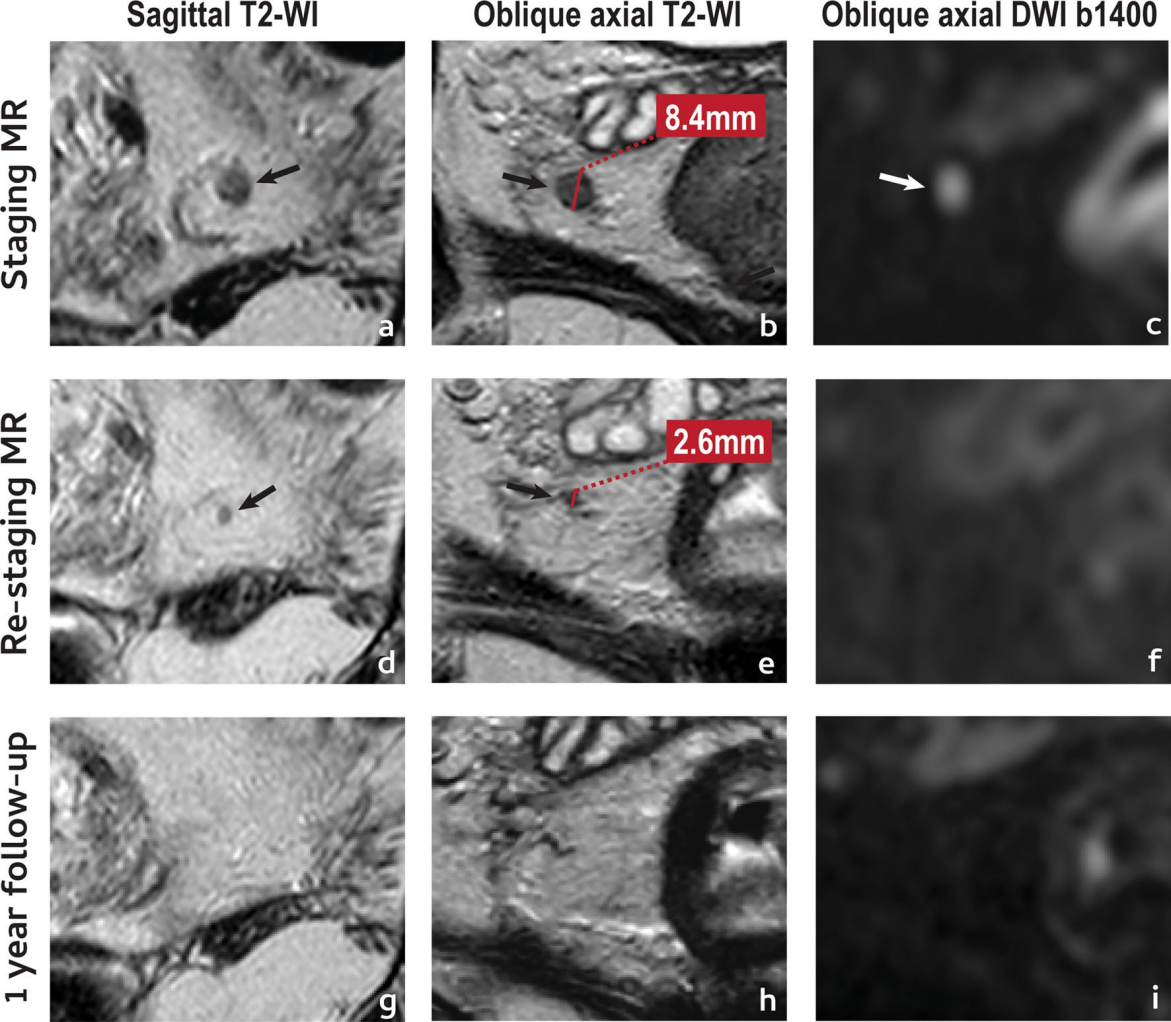
Complete lymph node response. A 42 year-old male presented with a low mrT3a (not shown) mrN1a (arrow in **a**–**c**) EMVI− CRM+ rectal cancer. He underwent NAT and was re-staged. The positive lymph node showed a pronounced reduction in size after NAT, with a short axis reduction of 70% and a short axis < 5 mm (**e**). On T2-WI, the lymph node became too small to characterise (**e**). The lymph node was not visible on re-staging high b value DWI (**f**) and disappeared also on T2-WI at 1-year follow up MR imaging (**g**–**i**). Patient is currently under W&W with no clinical signs of viable local or distant disease

An example of a complete lymph node response is provided in Fig. 7.

Lateral pelvic sidewall lymph nodes that shrink to 4 mm or less in short axis on re-staging MR imaging present no risk of local recurrence at 3 years according to Ogura et al. [51]. Disappearance or homogenous hypointensity on T2-WI and no visibility on high b value DWI may also favour a complete response.

Regarding response of extramural venous invasion, as stated above, mrV-TRG grades 1–3 (50% fibrosis or more) are associated with a good response, with only 9% LR rate and 88% 3-year DFS [39]. Even without concrete data on the subject, it appears reasonable to consider that normalisation of vessels or conversion to hypointense “fibrotic” signal intensity on T2-WI without high signal intensity on DWI would favour a complete response of extramural venous invasion, the same applying to extranodal tumour deposits.

Complete responders may be offered the possibility of entering a specialised surveillance program for organ preservation.

### To identify the “near-complete” responders

The criteria presented above are very specific but not very sensitive for a complete response. The concept of “near-complete response” was introduced more recently, driven by the observation that a significant proportion of patients presenting with a very good but incomplete response at first assessment may convert into a cCR if given a longer interval and re-assessment [52]. This concept is, however, controversial. The scarce literature regarding this group of patients considers that they may present with endoscopic gradings 1–2 and/or up to high-grade dysplasia at histopathology when biopsy is performed [52–54]. However, given a negative biopsy is not equivalent to a complete clinical response, particularly in the presence of clinically residual abnormalities, its utility may be put to question [55]. Regarding the MR criteria (Fig. 1d–f):

#### mrTRG2-2/3

Patients with dense fibrosis or with dense fibrosis and minimal residual intermediate signal may be considered near-complete responders [52, 53].

#### Small focal area of high signal intensity on high b value DWI

A small focal area of high signal intensity on high b value DWI is admissible for a near-complete response [53].

There is no evidence regarding expected tumour size reduction for these patients.

**Fig. 8:**
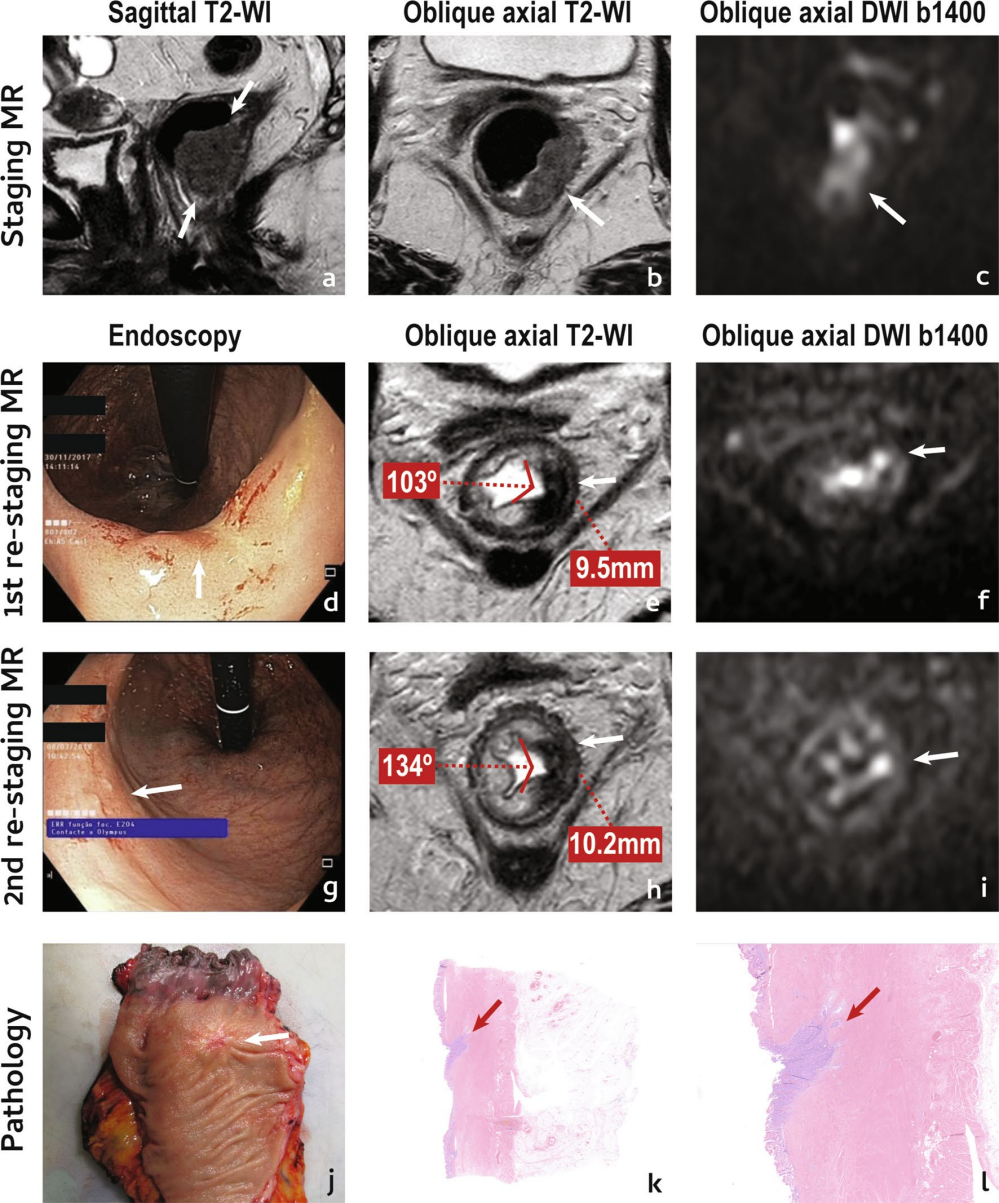
“Near-complete” tumour response. An 83 year-old female presented with a low mrT2 (arrows in **a**, **b**) mrN1a (not shown) EMVI− CRM− rectal cancer. She underwent NAT and was re-staged on endoscopy as grade 1 due to the presence of a red scar (arrow in **d**), and on MR imaging as mrTRG2/3 given tumour bed was now a black scar with a single focus of intermediate signal intensity below endoluminal aspect, a bit displaced from the centre (arrow in **e**), that presented with high signal intensity at high b value DWI (arrow in **f**). Patient was considered a “near-complete” responder and was followed. At 2nd re-staging, an adenomatous mucosal abnormality was observed at the periphery of the scar (arrow in **g**) and patient was considered a grade 2 on endoscopy. On MR imaging, she was now an mrTRG3 due to the expansion of the intermediate signal focus (arrow in **h**), which was also more conspicuous on high b value DWI (arrow in **i**). Patient underwent abdomino-perineal excision and specimen was a ypT3 ypN0 ypEMVI-TRG3 R0. The endoluminal regrowth was apparent on the gross examination of the fresh specimen (arrow in **j**) and at histology was depicted as a focal persistent niche of tumour at the endoluminal aspect of the tumour bed (arrows in **k** and **l**) growing in depth. It reached the mesorectal fat which was focally invaded (not shown)

An example of a near-complete tumour response is provided in Fig. 8.

Regarding lymph node involvement, the only study on near-complete response mentioning lymph nodes considers “suspicious” lymph nodes, whether mesorectal or sidewall, should not be present on re-staging MR imaging [53]. As such, the *same criteria as for complete responders* should be applied (Fig. 3d–f). Although again no data was found on the matter, the same may work for EMVI and extranodal tumour deposits.

## Follow-up of patients who deferred surgery

### Question 1: Can we anticipate a clinical complete response is going to be sustained?

There are some signs at re-staging MR imaging that, although not validated, may favour a sustained complete response:

1. *Normalisation of the rectal wall* at re-staging MR imaging has a 100% specificity for a sustained complete response according to the pattern base approach by Lambregts et al. [32].
2. *A positive split scar sign* at first re-staging MR imaging may indicate a sustained complete response for a minimum period of 1 year with a specificity of 97%, as per our data [49] (Figs. 5, 6).
3. *Hypointense “fibrosis” on T2-WI without high signal intensity on high b value DWI in semicircular tumours* is, according to Lambregts et al., associated with a sustained complete response with a 91% specificity [32].

### Question 2: When we observe a clinical complete response, can we anticipate a local regrowth is going to occur later on?

There are some signs at re-staging MR imaging that, although not validated, may associate with a higher likelihood of a local regrowth:

1. Tumour scar depth angle increase > 21° between 1st and 2nd post-NAT MR imaging

**Fig. 9:**
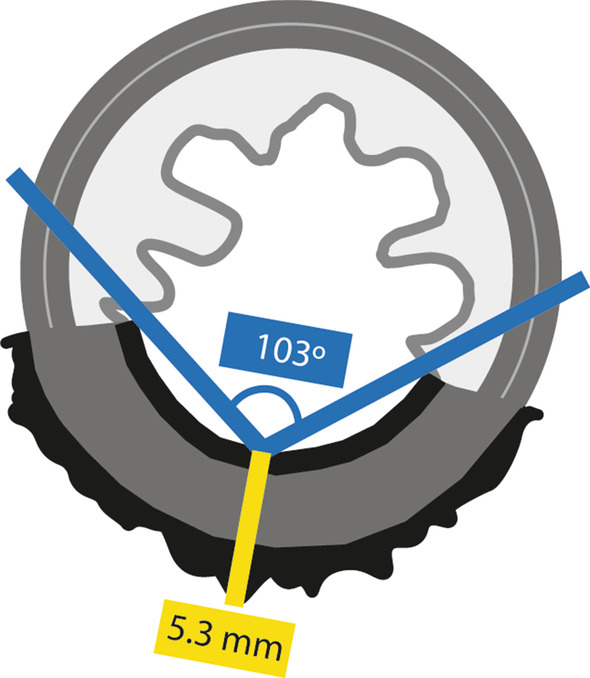
Scar depth angle and thickness measurements should be performed on the axial central slice of the tumour. For scar depth angle measurements of small (< 180°) tumour scars, the endoluminal centre of the scar serves as pivot while the angle lines cross the endoluminal extremities of the scar (in blue). In > 180° scars, the pivot should also be central but the angle lines should cross the endoluminal aspect of the scar at 180° of the rectal circumference and no more than that. The same applies to circumferential scars but in such cases, the pivot should be placed at the most invasive tumour front if there is one, or randomly if there isn´t. The most important thing to keep in mind is measuring at the exact same location in both first and second post-NAT MR imaging examinations. Scar thickness should be measured at pivot level, at the second MR imaging assessment. Measurement corresponds to a line that transverses tumour scar in depth, perpendicularly to it (in yellow), from inner to outer surface, irrespectively of it signal intensity

The scar depth angle is a measure of tumour bed contraction/dilation over time on T2-WI. For patients who enroll W&W, a scar depth angle increase of 21° or more between the first re-staging MR imaging examination (median 10 weeks post-RT) and the following (median 23 weeks post-RT) signaled a non-sustained complete response with a very high specificity (91/94%) [56]. To measure it, the central axial slice of the tumour must first be chosen using sagittal/coronal planes as reference. Then, within the central axial slice, the endoluminal centre of the scar should serve as pivot while the angle lines cross the endoluminal extremities of the scar (Fig. 9). For scars taking more than 180º of the rectal wall, the angle lines should cross the endoluminal aspect of the scar at 180º. The same applies to circumferential tumours, in which case the pivot should be placed at the endoluminal aspect of the point where it is most invasive (or randomly, if there is none). The most important thing to keep in mind is to keep the exact same measurement location between examinations. Figure 8 provides an example of tumour depth angle measurements and their significance—residual tumour focus grows into the lumen of the scar at second assessment, elevating pivot for angle measurement. Although it creates a sulc beside it, the scar as a whole “opens up”. Please note that these results come from a single institution and have not yet been validated prospectively [56].

2. Scar thickness > 10 mm at 2nd MR imaging assessment.

For patients who enroll W&W, a scar thickness > 10 mm in the first follow-up examination (median 23 weeks post-RT) was > 90% specific for a non-sustained complete response, irrespective of other findings, according to the results of the same study as above [56]. Tumour scar thickness measurement should be performed at pivot level and corresponds to the in-depth measurement of the tumour bed, perpendicularly to it, from its inner to outer surface (Fig. 9) (Case in Fig. 8 also presents with scar thickness > 10 mm at 2nd assessment).

### Question 3: How do we spot a rectal local regrowth?

In upfront clinical complete responders, we may expect a bit of scar “contraction” and/or scar “thinning” over time [56] but overall, patient preparation and acquisition technique assured, stable MR imaging findings are good MR imaging findings. Any change, even if subtle, should be reported but we should always make sure, before interpreting the findings, that no endoscopic procedures like biopsy, mucosectomy or local excision were performed given their potential false positive results. Subtle changes may include:

1. *Scar thickening* Scar thickening may be the first hint to a local regrowth and may present before any other MR imaging or endoscopic signs of macroscopic tumour [56].
2. *Depth angle increase* Depth angle increase may also be present before any obvious signs of a local regrowth on MR imaging or endoscopy and in fact 1. and 2. may be observed together [56].

More obvious changes indicating a local regrowth are:

3. *Intermediate “tumour” signal intensity/heterogeneity “*de novo*” at tumour scar on T2-WI* An mrTRG of 5 should be given when intermediate “tumour” signal appears de novo at tumour bed on W&W follow-up.
4. *High signal intensity at high b value DWI “*de novo*”* A bright spot or area de novo at tumour bed may indicate tumour regrowth with high specificity at it is particularly important when observed in depth, because it will be out of the scope of endoscopy.

### Question 4: How do we spot an extra-rectal local regrowth?

Extra-rectal local regrowth is uncommon (3%) [55]. Its early detection, whether in lymph nodes, extra-nodal tumour deposits or EMVI, is dependent on careful comparison with previous examinations.

1. *Conformation change*, such as apparently “sterilised” lymph nodes/tumour deposits that become rounder or more irregular.
2. *Increase in size*, which may be subtle and require zoomed-in measurement in multiple planes.
3. *Intermediate “tumour” signal intensity/heterogeneity on T2-WI *de novo*.*
4. *Focus/foci of high *signal* intensity on high b-value DWI *de novo*.*

**Fig. 10:**
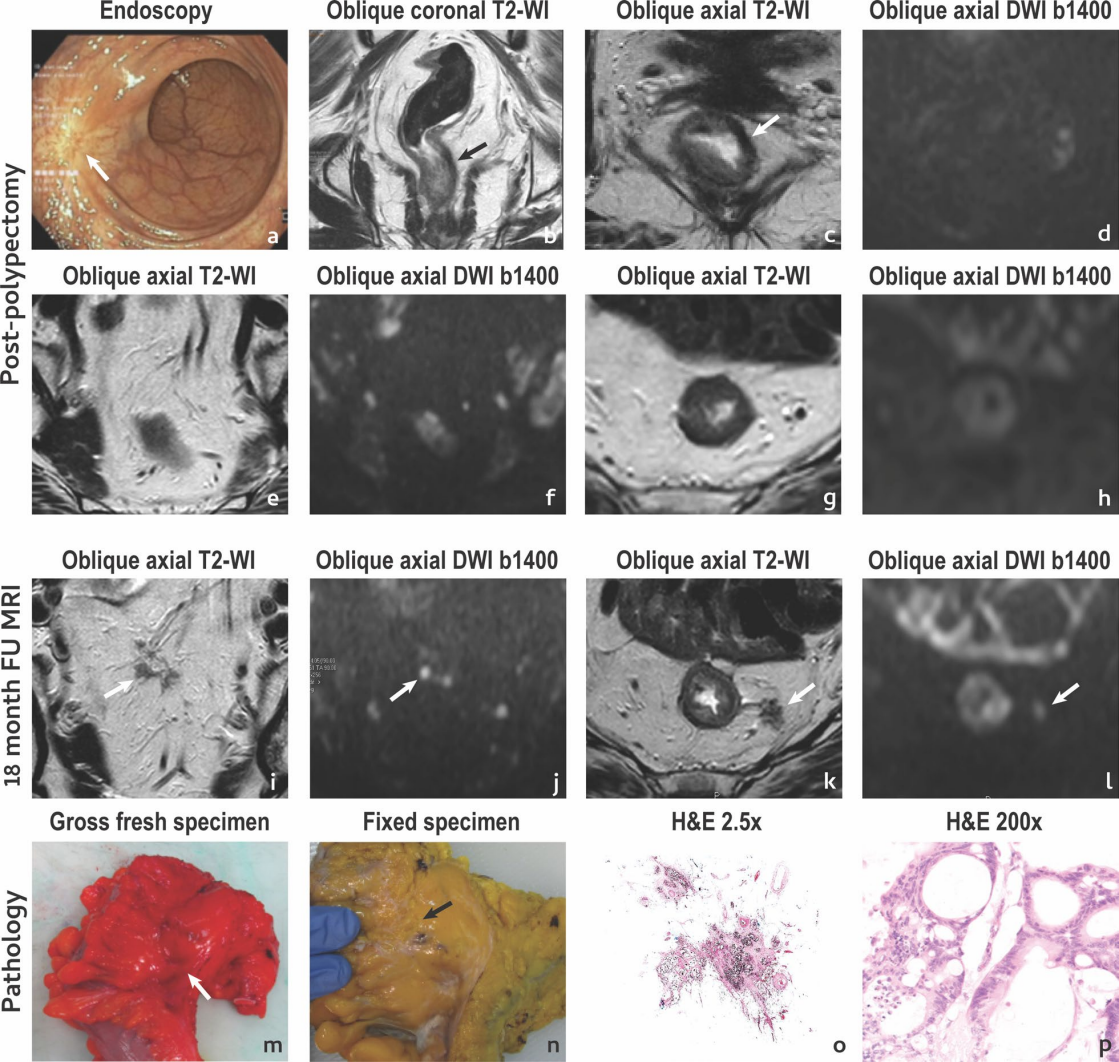
Extra-rectal pelvic recurrence. A 62 year-old female with rectal bleeding presented with a 34 mm polyp at colonoscopy which was excised revealing a tubulovillous adenoma with moderately differentiated adenocarcinoma, mucinous type, invading the muscularis propria with a focally positive margin in depth. No residual tumour was apparent on MR imaging or endoscopy at post-polypectomy assessment (**a**–**d**) and no extra-rectal suspicious findings were found either (**e**–**h**). Patient underwent long course chemoradiation and was followed. Findings were stable until the 18th month of follow-up, when irregular, heterogeneous, intermediate signal foci were found cranially to the polypectomy scar, within the mesorectal fat, at two different levels (arrows in **i**, **j** and **k**, **l**). Patient underwent total mesorectal excision and the MR imaging findings corresponded to extranodal tumour deposits of intestinal type adenocarcinoma with mucinous areas. The extranodal tumour deposits in **i**, **j** are shown in the fresh (**m**) and fixed (**n**) pathology specimen and at hematoxilin eosin staining both at low (× 2.5) (**o**) and high (× 200) (**p**) magnification

It is important to state that not all extra-rectal pelvic recurrences are clear regrowths. Uncommonly, disease may emerge in lymph nodes classified as innocent upon staging or as extranodal tumour deposits in a location in which only fat/vessels were present before, so carefull evaluation of the whole pelvis is imperative (Fig. 10).

### Question 5: Upon conversion of a “near-complete” response to a clinical complete response, is patient prognosis the same compared to upfront clinical complete responders?

It is important to state that the management for this group of patients is controversial. In the study by Simpson et al. [53], 63% of the “near-complete” responders evolved to a cCR within a median time of 8.5 months. Compared to upfront clinical complete responders, there was an increase in the local regrowth rate from 14 to 18% and the disease-free survival dropped from a median 60.5 to 33 months [53]. Hupkens et al. [52] reported a 90% conversion of “near-complete” responders at first assessment to a clinical complete response if given 6–12 additional weeks, but with an increase in local regrowth rate from 15.9 to 27.1%. No impact on OS was reported in either studies [52, 53]. The incidence of distant metastasis is higher in patients with local regrowth (17.8%) than in sustained complete responders (4.9%) [55]. In summary, late complete responses may come at the cost of a higher rate of local regrowths, which in turn may be associated with a higher incidence of metachronous metastases. Whether the latter result from upfront differences in tumour biology or as a consequence of an uncontrolled primary is not yet known.

## Re-staging and follow-up reporting template

**Fig. 11:**
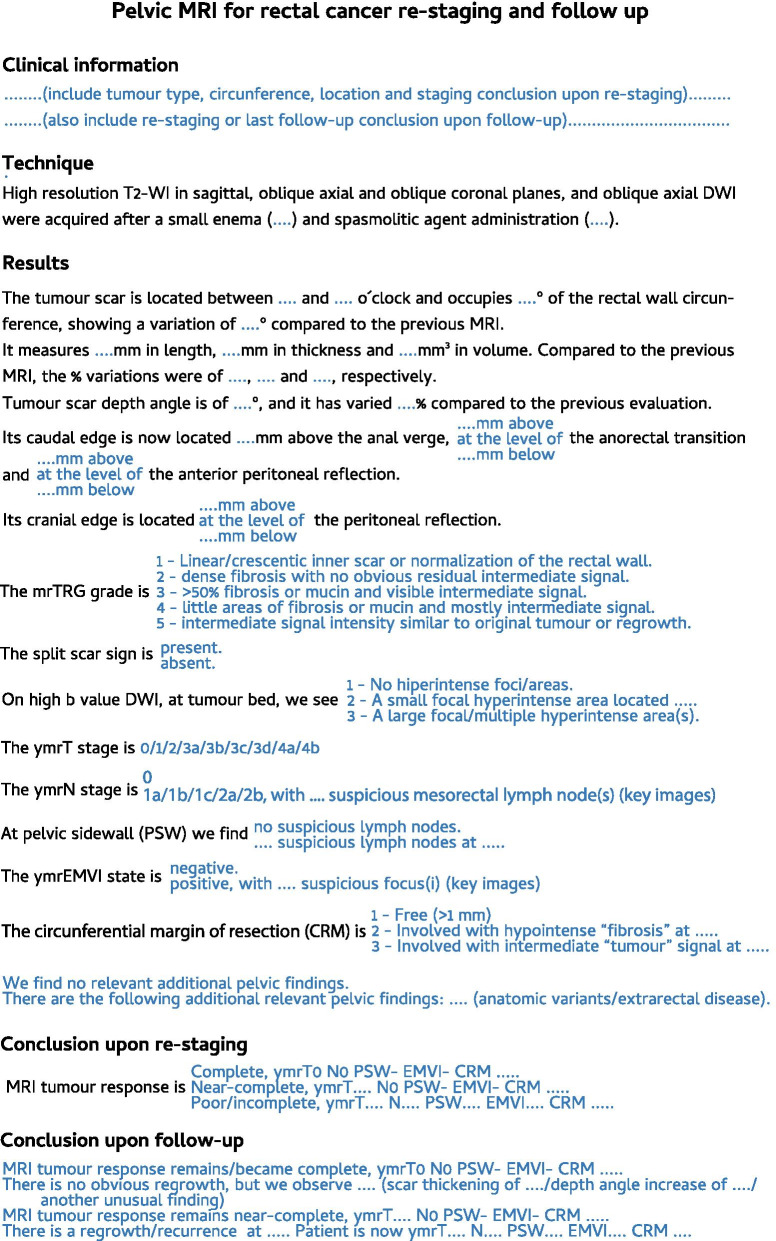
Re-staging and follow-up report template. Our proposed standardised report template is depicted. In blue, a single option should be chosen

Our proposed re-staging and follow-up reporting template is shown in Fig. 11. Please keep in mind that it is a mere suggestion and has not been validated in a prospective and multi-institutional setting.

Compared to the re-staging report template provided by ESGAR [10], ours is more complex, time-consuming and cumbersome to use because it includes multiple methods reported for response assessment and also contemplates follow-up imaging after re-staging. It involves recording thickness, volume, depth angle and their variation compared to the previous examination, given its potential, unvalidated utility in the early prediction of a non-sustained complete response, as discussed above. Also, our response assessment of the primary tumour is based on separate mrTRG, split scar sign and 3-point ordinal scale DWI evaluation, while ESGAR recommends using a 3-point combined T2-WI/DWI ordinal scale.

## Conclusion

“Watch-and-Wait” rectal cancer programs are growing around the world and revolve around endoscopy and pelvic MR imaging, both for re-staging and patient follow-up. Radiologists involved in such programs should be familiar with the imaging findings that suggest a poor/incomplete response, a complete response or a “near-complete” response and their prognostic implications. They should also be equipped for the early detection of local regrowths, in depth at tumour bed or extra-rectal in particular, given their invisibility at rectoscopy.

## Data Availability

Not applicable.

## Abbreviations

CRM: Circumferential resection margin
DWI: Diffusion-weighted imaging
EMVI: Extramural venous invasion
MR: Magnetic resonance
mrTRG: Magnetic resonance tumour regression grade
mr-vTRG: Magnetic resonance venous tumour regression grade
NAT: Neoadjuvant therapy
pCR: Pathologic complete response
RT: Radiotherapy
W&W: Watch and Wait
